# Capecitabine Monotherapy as Palliative Treatment for Patients with Recurrent/Metastatic Nasopharyngeal Cancer

**DOI:** 10.1155/2022/6860413

**Published:** 2022-04-05

**Authors:** Bader Alshamsan, Ahmed Mostafa Gad, Maaz Kamal Alata, Mashari Alzahrani, Tusneem Elhassan, Jean Paul Atallah

**Affiliations:** ^1^Medical Oncology, Oncology Center, King Faisal Specialist Hospital and Research Center, Riyadh, Saudi Arabia; ^2^Department of Medicine, Qassim Medical College, Qassim University, Qassim, Saudi Arabia; ^3^Clinical Oncology and Nuclear Medicine Department, Faculty of Medicine, Ain Shams University, Cairo, Egypt; ^4^Medical Oncology Department, Prince Mohamed Bin Naser Hospital, Gizan, Saudi Arabia

## Abstract

**Background:**

Numerous chemotherapeutic agents have antitumor activity in recurrent/metastatic (R/M) nasopharyngeal cancer (NPC). Evidence of capecitabine's effectiveness as monotherapy is limited. Capecitabine tolerability in solid malignancies has ethnic and geographical variability. We investigated capecitabine's tolerability and identified potential prognostic factors for clinical outcomes in R/M NPC.

**Methods:**

A consecutive retrospective cohort of patients who received capecitabine as the first recurrence, second- or third-line monotherapy for metastatic NPC (2011–2019) was reviewed concerning patient characteristics, pathological features, treatment outcomes, and toxicity.

**Results:**

Fifty-one patients were eligible (median age at diagnosis: 42 [35.5–52.5] years). Most patients (78.4%) tolerated a standard oral dose of 1,250 mg/m^2^ capecitabine (2 weeks on/1 week off) in a 3-week cycle. The objective response rate was 49%, and the disease control rate was 66.7%, with a median response duration of 6.2 months. Hand-foot syndrome (HFS) was associated with a higher objective response rate (odds ratio, 5.1 [95% confidence interval: 1.18–21.98]; *P* = 0.02). The median follow-up duration was 17.8 (interquartile range: 7.8–30.4) months. The median (95% confidence interval) progression-free survival and overall survival were 6.6 (4.3–8.8) and 32.7 (25.9–39.5) months, respectively. HFS (*P* = 0.02), better performance status (*P* = 0.02), and absence of brain metastasis (*P* = 0.04) were associated with prolonged progression-free survival.

**Conclusion:**

Capecitabine monotherapy is effective and well-tolerated as a palliative treatment for R/M NPC. Despite the lower incidence of HFS in our patients, it remained a favorable prognostic factor for objective response and progression-free survival.

## 1. Introduction

Nasopharyngeal carcinoma (NPC) is a rare malignancy responsible for approximately 0.7% of all new cancers diagnosed worldwide in 2020 [1]. The highest NPC incidence is observed in Asia, followed by Africa, and NPC is rarely diagnosed in Europe and North America [2]. In Saudi Arabia, NPC is more common in the central and northern parts of the country (e.g., Qassim and Jouf) [3]. The non-keratinizing histological subtype is prevalent in endemic regions and is associated with Epstein–Barr virus infection, with higher sensitivity to chemotherapy and radiotherapy [4, 5].

The recommended regimen for primary definitive treatment is concurrent chemoradiotherapy plus (neo)adjuvant cisplatin-based chemotherapy [6]. Cisplatin/gemcitabine is the first-line therapy for recurrent or metastatic disease (R/M) [6–8]. Most patients with R/M NPC have already exhausted platinum-based therapy, and there is no substantial evidence to support the choice of treatment in subsequent lines [9]. Palliative combination therapy uses various effective agents that show significant treatment benefits; however, toxicity is significantly high [10–14]. Single-agent chemotherapy, such as gemcitabine [15], paclitaxel [16], irinotecan [17], methotrexate [18], and capecitabine [19, 20], is a viable option for the management of R/M NPC. Immunotherapy also demonstrated moderate efficacy in non-randomized trials of patients with pretreated R/M NPC.[21–23]

Capecitabine monotherapy in NPC was evaluated initially in China in a phase II trial of 17 patients [19], then the same author reported the outcomes of 49 patients in a retrospective study [20]. The objective response rate (ORR) was 23.5% and 37%, respectively. Ethnic and geographical differences in the tolerability of capecitabine were observed with other solid malignancies [24]. Studies comparing capecitabine usage in Arab or Middle Eastern ethnicity are lacking. This study aimed to evaluate the efficacy and tolerability of capecitabine in patients with R/M NPC and to identify potential prognostic factors for clinical outcomes.

## 2. Materials and Methods

The electronic medical records of a consecutive cohort of patients who received capecitabine as a single-agent treatment for R/M NPC between April 2011 and August 2019 at the King Faisal Specialist Hospital and Research Center, Riyadh, were reviewed. Ethical approval was obtained from the Research Approval Committee (RAC# 2191100), and the requirement for patient informed consent was waived. The data obtained included patient characteristics, performance status, baseline laboratory test results, staging, pathological features, treatment lines, starting dose of capecitabine, dose modifications, best responses, toxicity profiles, and time to progression.

The Eastern Cooperative Oncology Group Performance Status (ECOG-PS) assessment was used, and tumors were staged according to the American Joint Committee on Cancer Union for International Cancer Control Tumor–Node–Metastasis staging system.

The standard dose of capecitabine in our institution is 1,250 mg/m^2^ twice daily for 2 weeks, followed by a 1-week rest in 3-week cycles. However, the starting dose could be 850 mg/m^2^ or 1000 mg/m^2^ and escalate to the standard dose if tolerated well. All patients underwent baseline staging with magnetic resonance imaging (MRI) of the head and neck, computed tomography (CT) of chest, abdomen, and pelvis, and bone scan or positron emission tomography (PET) CT scans.

The patients were assessed before the second cycle in the oncology clinic, and prechemotherapy assessment clinic for clinical evaluation and revision of laboratory work, including complete blood counts, renal and hepatic profiles, and management of treatment-related toxicity, were done if needed. Treatment-related toxicity was evaluated according to the Common Terminology Criteria for Adverse Events (version 4.0).

The objective responses were assessed every three cycles with MRI of the head and neck, chest abdomen, and pelvis CT scan; bone scan, or PET CT where appropriate, using the Response Evaluation Criteria in Solid Tumors (version 1.1). All patients were assessed for clinical outcomes, including ORR, progression-free survival (PFS), and overall survival (OS). The date of the last follow-up was September 2021.

ORR was defined as the sum of the percentage of patients who had partial or complete responses after starting capecitabine. The disease control rate (DCR) comprised complete response, partial response, and stable disease. The duration of response to capecitabine was defined as the time from confirmation of partial response, complete response, or stable disease until disease progression or death. PFS was defined as the time from the beginning of capecitabine therapy until disease progression or death. The time from the beginning of capecitabine therapy until death from any cause was defined as OS.

Categorical and continuous variables were described as frequencies and median with interquartile ranges (IQR), respectively. The significance of the predictors was estimated using logistic regression. The ORR to capecitabine was considered the main effect maintained in the model. The interaction between ORR and capecitabine and other significant variables was evaluated. Subsequently, a stepwise selection process was performed at *P* = 0.10 for entering the model and *P* = 0.05 for remaining in the model; all parameters with *P* = 0.25 in the univariate analysis were considered. The Kaplan–Meier method was used to estimate PFS and OS. Survival curves were compared using the log-rank test and Cox regression analysis. A *P*-value <0.05 was considered statistically significant, and statistical analyses were performed using SPSS (version 27) (IBM Corp., Armonk, NY, USA).

## 3. Results

### 3.1. Patients and Disease Characteristics

Fifty-one patients were eligible for this study, with a median age at diagnosis of 42 years (IQR: 35.5–52.5). The majority (98%) had non-keratinized undifferentiated squamous cell carcinoma and (97.2%) were Epstein–Barr virus-positive. In this cohort, 52.9% of patients had an initial presentation with localized disease and then developed recurrence; 47.1% presented with *de novo* metastases. Patient and disease characteristics are presented in Table 1. There was no prior use of fluoropyrimidine in this cohort. Capecitabine was used as the first-, second-, or third-line treatment in 21.6%, 35.3%, and 43.1% of the patients, respectively. Twenty-four patients received subsequent therapy after capecitabine treatment: gemcitabine [15], taxane [3], platinum [3], and immunotherapy [3]. Data for response and tolerability to capecitabine were available for all patients, and eight patients were lost to follow-up (15.6%).

### 3.2. Capecitabine ORR and Predictive Variables

ORR and DCR were 49% and 66.7%, respectively. The median duration of capecitabine treatment was 6.5 (IQR: 4.2–15.6) months, and the median duration of response was 6.2 (IQR: 3.3–13.5) months.  Table 2 shows the best responses to capecitabine.

The patients with hand–foot syndrome (HFS) showed a higher ORR than those without (*P* = 0.02). In addition, a history of smoking was associated with a lower ORR (*P* = 0.02). However, data regarding smoking history were missing for 29.4% of the patients, and the active smoking status during therapy was unknown. There was no association between HFS and starting capecitabine with a lower dose *vs*. standard dose (*P* = 0.27) or with the highest achieved capecitabine dose (standard *vs*. lower dose) (*P* = 0.91).  Table 3 presents the logistic regression analysis of the variables associated with the ORR to capecitabine in R/M NPC. The adjusted odds ratio for capecitabine dose showed that the development of HFS remained significant for the ORR (OR: 5.1 [95% CI: 1.18–21.98]; *P* = 0.02).

### 3.3. Survival Analysis

**Figure 1:**
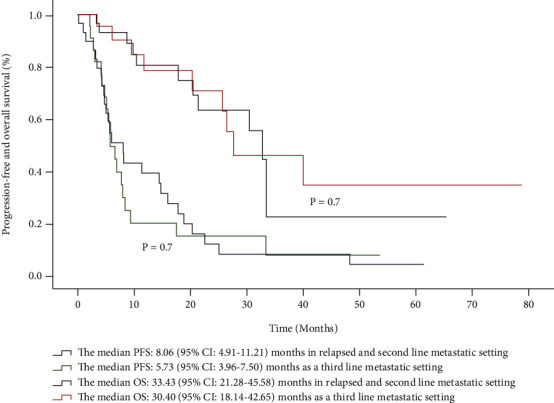
Kaplan–Meier curves of median progression-free survival and overall survival in patients with nasopharyngeal cancer stratified by order of capecitabine treatment.

The median follow-up was 17.8 (IQR: 7.8–30.4) months. The median PFS was 6.6 (95% CI: 4.3–8.8) months, and the median OS was 32.7 (95% CI: 25.9–39.5) months; the order of using capecitabine (second-line *vs*. third-line) did not correlate with differences in PFS (*P* = 0.7) or OS (*P* = 0.7) (Figure 1).

**Figure 2:**
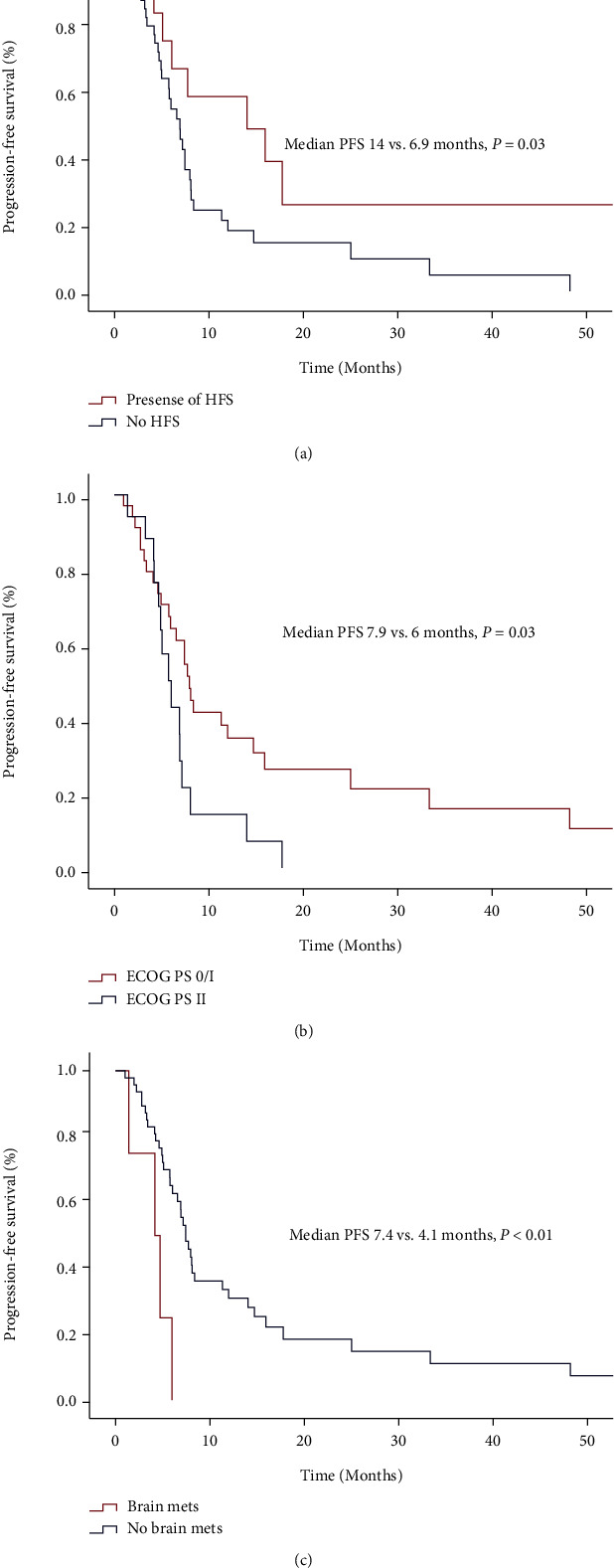
Kaplan–Meier curves of progression-free survival in patients with nasopharyngeal cancer treated with capecitabine stratified by hand-foot syndrome (a), Eastern Cooperative Oncology Group Performance Status (b), and the presence of brain metastasis (c).

The presence of HFS, ECOG-PS 0/1 *vs*. ≥2, and the absence of brain metastasis were associated with prolonged PFS (Figure 2(a)–2(c)). There were no significant differences in PFS with respect to sex (*P* = 0.33), history of smoking (*P* = 0.45), metastasis at diagnosis (*P* = 0.94), recurrence type (*P* = 0.49), bone metastasis (*P* = 0.70), lung metastasis (*P* = 0.98), liver metastasis (*P* = 0.97), or type of chemotherapy (first-line anthracycline [*P* = 0.48] or taxane [*P* = 0.75]). In the multivariate Cox regression analysis, HFS (hazard ratio [HR]: 0.35 [95% CI: 0.14–0.87]; *P* = 0.02), ECOG-PS (HR: 2.3 [95% CI: 1.10–4.87]; *P* = 0.02), and brain metastasis (HR: 3.2 [95% CI: 1.04–10.07]; *P* = 0.04) remained significant. There were trends for clinical differences in OS concerning HFS (median OS: 33.4 *vs*. 20.2 months) and absence of a history of smoking (median OS: 32.7 *vs*. 25.6 months). However, these differences were not statistically significant (*P* = 0.052 and *P* = 0.38, respectively).

### 3.4. Capecitabine Tolerability

Twenty-one (41%) patients started with a standard dose of 1250 mg/m^2^, and 30 patients began the first cycle with a reduced dose of either 850 mg/m^2^ (10, 16%) or 1000 mg/m^2^ (20, 43%) and then escalated to the standard dose with good tolerance. Most patients (78.4%) tolerated the standard oral dose of 1,250 mg/m^2^ twice daily for 2 weeks, followed by a 1-week rest in 3-week cycles. Twenty percent of patients required a 25% dose reduction, and one required a 50% dose reduction. The reasons for capecitabine discontinuation were disease progression in 44 (86.2%) patients, patient request in three (5.8%) patients, and toxicity in three (5.8%) patients. After the first cycle, one patient developed fatigue, another developed neutropenia, and a third developed HFS, leading to treatment discontinuation; detailed toxicity profiles are shown in Table 4.

## 4. Discussion

This was the first study of capecitabine monotherapy in R/M NPC from the Middle East, specifically Saudi Arabia. The efficacy was comparable to the reported outcomes of previous studies that used capecitabine as a single-agent treatment for R/M NPC. The ORR of 49% and DCR of 66.7%, with a median response duration of 6.5 months, were comparable to or better than the corresponding values reported in previous studies (Table 5). The higher median OS of 32.7% (95% CI: 25.9–39.5) months in this cohort in comparison to previous studies [25] cannot be attributed to capecitabine rather than to the patient and disease characteristics (e.g., median age of 42 years, ECOG-PS 0/1 > 66.7%), and other subsequent chemotherapy treatment lines (47%).

The results were also comparable to those pertaining to immunotherapy [21–23]. Pembrolizumab had an ORR of 26% and a median duration of response of 17.1 months. The 1-year OS rate was 63%, the 1-year PFS rate was 34%, and approximately 30% of patients experienced a grade III–V drug-related adverse event [22]. Nivolumab had an ORR of 20%, a 1-year OS rate of approximately 59%, and a 1-year PFS rate of 19.3% in two-phase I/II trials [21, 23].

KEYNOTE-122 showed that, compared to chemotherapy, pembrolizumab did not improve the ORR, PFS, or OS of patients with recurrent metastatic NPC [27]. However, the addition of immune checkpoint inhibitors (toripalimab or camrelizumab) to gemcitabine plus cisplatin improved PFS in two separate phase III trials of R/M NPC [28, 29].

Most patients (80%) in our study tolerated the standard dose of capecitabine. Only 20% of patients required a 25% dose reduction, and one required a 50% dose reduction. Ten patients (19.6%) developed grade III or IV toxicities, and no hospitalizations or deaths due to toxicity were reported. A landmark East Asian study by Chau *et al.* of capecitabine use for NPC treatment reported that 41% of patients required hospitalization, and 11% died of treatment-related causes [20]. The incidence of toxicity in our patients was much lower than that reported in a previous study from China. This variation could be related to other factors, such as age at diagnosis, body weight, ECOG-PS, genetic polymorphisms associated with ethnicity, psychosocial factors, and dietary folate intake [30]. Recently, adjuvant therapy with capecitabine (650 mg/m^2^) after definitive chemoradiotherapy was shown to have high tolerability and a low toxicity profile in a Chinese study [31].

Our study confirmed that developing HFS is associated with a better objective response, as reported in a previous study. However, the incidence of HFS was lower in our patients (27.4% *vs*. 86%) [20]. HFS was first reported in 1984 with 5-fluorouracil [32], and later with cytarabine, doxorubicin, capecitabine, and other chemotherapeutic and biologic agents [33]. Development of HFS during capecitabine therapy has been associated with better outcomes in other cancers, e.g., colon [24], head and neck [19], and metastatic breast cancer [34]. Furthermore, a meta-analysis of more than 4,700 patients treated with capecitabine alone or in combination with other agents for solid cancers showed that patients who developed HFS had longer OS (36.1 *vs*. 22.7 months) (HR: 0.61 [95% CI: 0.56–0.66]) [35]. Serum folate (*P* < 0.001) and red blood cell folate (*P* = 0.001) levels were reported as predictors of grade II or higher HFS.^36^ HFS could be used as an encouraging prognostic variable of response to capecitabine therapy while treating patients with NPC.

Despite the limitation that smoking history data were missing for 29.4% of patients and active smoking status during therapy was unknown, we observed that the ORR was substantially higher among non-smokers, a finding that requires further research to validate the prognostic value of smoking.

We acknowledge the limitations of a retrospective study, which decreased the certainty of prognostic markers, such as smoking history, and the accuracy of non-hematological toxicity profiles and quality-of-life outcomes. Moreover, this study had a small sample size. However, the sample size was slightly larger than that reported in previous studies.

## 5. Conclusion

Capecitabine is an effective and well-tolerated chemotherapeutic agent conveniently used as a monotherapy for palliative treatment of R/M NPC. HFS was predictive of objective response and associated with better PFS. Further trials are required to obtain substantial evidence to support these findings.

## Figures and Tables

**Table 1:** Patients and disease characteristics (*n* =51).

Characteristics	Number/median (frequency/IQR)
Age at diagnosis	42 (35.5–52.5)
Sex	
Male	39 (76.5)
Female	12 (23.5)
ECOG-PS before starting capecitabine	
0/1	34 (66.7)
2	17 (33.3)
History of smoking	
Present	8 (15.7)
Absent	28 (54.9)
NA	15 (29.4)
Stage at initial diagnosis	
I	0
II	1
III	5 (9.8)
IVA	21 (43.1)
IVB	24 (47.1)
Metastatic sites	
Lung	28 (54.9)
Liver	17 (33.3)
Bone	24 (47.1)
Brain	4 (7.8)
Recurrence sites	27 (52.9)
Local only	4 (7.8)
Distant	15 (29)
Both	8 (15.7)
Prior chemotherapy	51 (100)
First-line:	
Platinum-based	51 (100)
With taxane	21 (41.1)
With anthracycline	27 (53)
With gemcitabine	3 (5.9)
Second-line:	
Platinum-based	17 (33)
With taxane	15 (29.4)
With gemcitabine	2 (3.9)
Monotherapy	23 (45)
Taxane	19 (37.2)
Platinum	3 (5.9)
Gemcitabine	1 (1.9)
Radiation therapy	
Concurrent chemoradiotherapy	47 (92)
Reirradiation	20 (39.2)
Capecitabine used as	
First recurrence	11 (21.6)
Second-line	18 (35.3)
Third-line	22 (43.1)

Abbreviations: ECOG-PS: Eastern Cooperative Oncology Group Performance Status; HFS: hand-foot syndrome; IQR: interquartile range.

**Table 2:** Best response to capecitabine.

Response	Number (%)
Complete response	8 (15.7)
Partial response	17 (33.3)
Stable disease	9 (17.6)
Progressive disease	17 (33.3)
ORR	25 (49)
DCR	34 (66.7)

Abbreviations: DCR: disease control rate; ORR: objective response rate.

**Table 3:** Regression analysis of variables associated with an objective response rate of capecitabine in R/M NPC.

Variables	Univariate analysis		
	OR	95% CI	*P*-value
ECOG-PS 0/1 *vs*. 2	1.26	0.39–4.06	0.69
De novo metastases *vs*. relapsed	0.67	0.22–2.04	0.48
Sites of metastasis			
Lung *vs*. others	1.07	0.35–3.23	0.89
Liver *vs*. others	0.80	0.24–2.53	0.69
Bone *vs*. others	0.67	0.22–2.04	0.49
Brain *vs*. others	0.29	0.02–3.02	0.30
1^st^/2^nd^ line *vs*. subsequent lines	1.07	0.35–3.24	0.90
Starting dose	0.41	0.13–1.28	0.12
Lower *vs*. standard dose ^a^			
Highest achieve dose			
Standard dose *vs*. lower dose	1.32	0.34-5.06	0.67
History of smoking *vs*. none	0.13	0.02–0.8	0.02
Development of HFS *vs*. none	5.5	1.29–23.3	0.02

^a^Lower starting dose are 850 mg/m^2^ or 1000 mg/m^2^ and standard dose is 1250 mg/m^2.^ Abbreviations: CI: confidence interval; ECOG-PS: Eastern Cooperative Oncology Group Performance Status; HFS: hand-foot syndrome; NPC: nasopharyngeal carcinoma; OR: odds ratio; R/M: recurrent/metastatic.

**Table 4:** Treatment-related toxicity of capecitabine.

Adverse event	Any grade	Grade I/II	Grade III/IV
Hand-foot syndrome	14 (27.4)	11 (21.5)	3 (5.9)
Fatigue	9 (17.6)	8 (15.7)	1 (2)
Diarrhea	8 (15.6)	8 (15.6)	
Nausea/vomiting	7 (13.7)	4 (7.8)	3 (5.9)
Mucositis	2 (3.9)	2 (3.9)	
Hematological			
Anemia	5 (9.8)	5 (9.8)
Thrombocytopenia	4 (7.8)	4 (7.8)
Neutropenia	3 (5.8)	2 (3.9)	1 (2)
Elevation of liver transaminases	2 (3.9)	1 (2)	1 (2)

Abbreviations: HFS: hand-foot syndrome. Data are presented as numbers (percentages).

**Table 5:** Summary of capecitabine trials of recurrent/metastatic NPC.

Author, year	Region	Number of patients	Capecitabine protocol	ORR	mPFS	mOS
Chua et al., 2003 [19]	Asia	17	1,250 mg/m^2^ BID for 2 weeks, Q3 weeks	23.5	4.9	7.6
Chua *et al.*, 2008 [20]	Asia	49	1,000–1,250 mg/m^2^ BID for 2 weeks, Q3 weeks	37	5	14
Ciuleanu *et al.*, 2008 [26]	Europe	23	2,500 mg/m^2^ BID for 2 weeks, Q3 weeks, to a maximum of six cycles	48	14	NR^a^
Current study, 2022	Middle East	51	1,250 mg/m^2^ BID for 2 weeks, Q3 weeks	49	6.6	32.7

^a^Not reached at 18 months and 1-year OS was 62%. Abbreviations: BID: twice a day; mOS: median overall survival; mPFS: median progression-free survival; NPC: nasopharyngeal carcinoma; ORR: objective response rate; Q3 weeks: every 3 weeks.

## Data Availability

The datasets used and/or analyzed during the current study are available from the corresponding author on reasonable request.

## Abbreviations

CI: Confidence interval
ECOG-PS: Eastern Cooperative Oncology Group Performance Status
HFS: Hand-foot syndrome
HR: Hazard ratio
IQR: Interquartile range
NPC: Nasopharyngeal carcinoma
OR: Odds ratio
ORR: Objective response rate
OS: Overall survival
PFS: Progression-free survival
R/M: Recurrent/metastatic.
